# A new single switch universal supply voltage DC-DC converter for PV systems with MGWM-AFLC MPPT controller

**DOI:** 10.1038/s41598-024-62171-3

**Published:** 2024-05-27

**Authors:** Ezzeddine Touti, Shaik Rafikiran, Mouloud Aoudia, Ibrahim Mohammed Alrougy, Baseem Khan, Ahmed Ali

**Affiliations:** 1 Department of Electrical Engineering, College of Engineering, Northern Border University, Arar, 73222 Saudi Arabia; 2 Department of Electrical and Electronics Engineering, Sri Venkateswara College of Engineering, Tirupati, 517507 Andhra Pradesh India; 3 Department of Industrial Engineering, College of Engineering, Northern Border University, Arar, 73222 Saudi Arabia; 4 King Abdulaziz City for Science and Technology (KACST), Riyadh, Saudi Arabia; 5 Department of Electrical and Computer Engineering, Hawassa University, Hawassa, Ethiopia; 6 Department of Electrical and Electronic Engineering Technology, University of Johannesburg, Johannesburg, South Africa

**Keywords:** Active converter operation, Duty signal, Fast system response, Good dynamic behavior, High gain, Low design difficulty, Quick change of solar irradiations, Plus very flexible operation, Energy science and technology, Electrical and electronic engineering, Energy infrastructure

## Abstract

The present power generation government companies focus on Renewable Power Sources (RPS) because their features are zero carbon footprint, unlimited power source, fewer greenhouse pollutants, fewer output wastages, plus creatinga very healthy atmosphere. In this work, the sunlight source is utilized for the Photovoltaic (PV) standalone network. The merits of sunlight sources are very optimal human resources needed, unlimited natural sources, plus easy operation. However, the solar power resource is nonlinear fashion. As a result, the operating point of the sunlight network fluctuates concerning sunlight intensity. So, in this article, the Modified Grey Wolf Methodology with Adaptive Fuzzy Logic Controller (MGWM-AFLC) is introduced to maintain the operating point of the sunlight system at the global power point position of the PV array. This controller traces the MPP with very low fluctuations in the PV-produced voltage. The advantages of this proposed method arefewer sensing devices required, less difficulty in development, more useful for rapid changes inthe sunlight temperatures, simpler to realize operation, greater economic growth, plus highly useful for household applications. The sunlight set-up generation voltage is lowwhich is improved by introducing the new Wide Power Rating High Voltage DC-DC Boost Converter (WPRHVBC). The features of this WPRHV converter are low voltage strain on semiconductor devices, few passive elements are enough to develop the circuit, plus easy understanding.

## Introduction

In the present available power production scenario, the conventional power network usage is much less because its characteristics area time-consuming process for the production of power, more pollutants, high destructionof the natural ecosystem, more displacement in local communities, produces more hazardous gasses like CO_2_, NO_2_, and SO_2_. Also, its initial set-up price could be very high^1^. The available conventional power networks are coal systems, thermal energy, nuclear sources, geothermal, plus fuelwood. All these sources' initial development costs more, plus require high human power. So, the renewable power resources networks are focused on in article^2^ for energy supply to household applications. Solar, geothermal, tidal, wind, hydro station, plus bioenergy. The geothermal power supply is mainly contingent on the earth's surface water heating. In this system, the underground fluid is pumped to the surface of the earth to produce the heat. The steam turbines are interlinked in the geothermal network for the power distribution to the automotive systems. The merits of geothermal systems are reliable, no fuel is required, are highly sustainable, have huge potential, are environment friendly, plus rapid evaluation. However, this network's drawbacks are earth surface instability, highly expensive power transmission, plus the high possibility of depletion of geothermal sources.

The tidal network usage is improved in the coastal areas instead of geothermal because of its properties such asthe possibility of power supply at very low-level tidal speeds, more density of water when associated with the air, affordable cost to maintain, plus high fluently predictability. Tidal energy is one form of ocean kinetic energy^3^. Here, tides fall and rise kinetic energy is directly interfaced with the water turbine for achieving a small amount of useful power. The heavy tides come because of the gravitational interaction between the sun, moon, plus earth. This system is very efficient for converting 80% of tides into electrical power. In addition, it is highly reliable when associated with oil, natural gas, plus coal. However, the tidal networks are not applicable for large-scale power production. The wind systems are introduced in the article^4^ to limit the disadvantages of tidal systems. The wind turbines capture the wind velocity by utilizing the rotor propeller which is fed to the rotor shaft to produce the electricity. The wind plants are installed near the ocean, plus hilly regions for achieving the peak power from the wind blades. The main features of wind plants are zero CO_2_ release, simplicity in equipment development, simple operation, plus low effect on the atmosphere. In addition, it is a surface energy that is available everywhere, plus no worry about the absence of fossil fuel.

The drawbacks of wind blades are noise production, plus availed only remote locations. So, the fuel stack technology is interlinked with the wind/battery sources to improve the power supply reliability. In this hybrid power grid, fuel stack voltage is very low which is not acceptable for peak load demand. So, the power converter circuits are integrated with the wind networks for operating the entire system in a dual-direction power supply. However, the input fuel supply to the fuel stack is highly costly. So, the fuel source energy is not applied for emergency applications. However, the demerits of the above conventional, plus renewable systems are compensated by selecting sunlight energy. Solar is one of the popular available energies for battery charging systems. In Ref.^5^, the researchers combined the three renewable resources which are fuel stack/wind/solar systems. These are the power networks utilized by the different converter circuits for the hybrid renewable power network which are interleaved, bi-directional, plus high voltage conversion ratio converters. Here, each converter has its own merits and demerits, the interleaved circuit needed a high number of inductive components to enhance the power rating of the wind system. The bidirectional circuits are integrated with the sunlight system-dependent battery charging for maintaining the grid power factor unity. The cost of this overall circuit is more^6^.

**Figure 1:**
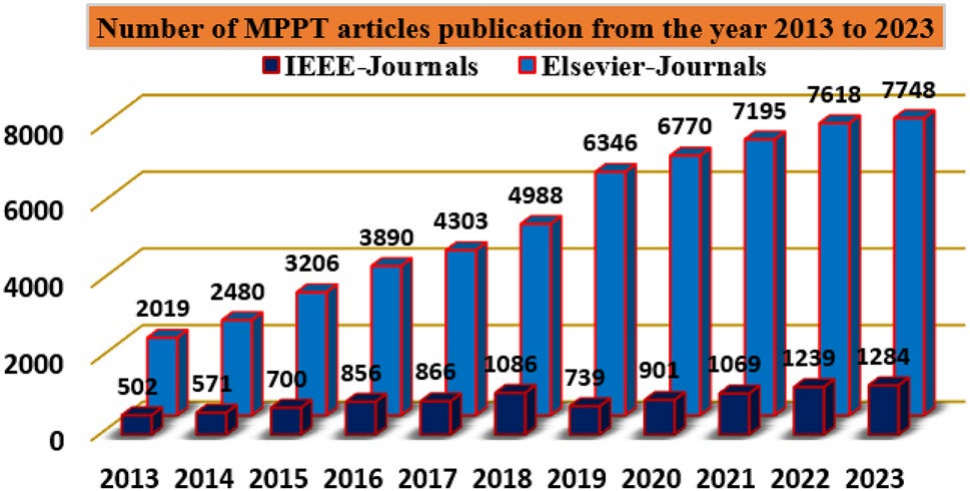
Strategy of power point identifiers for various sunlight intensity values.

**Figure 2:**
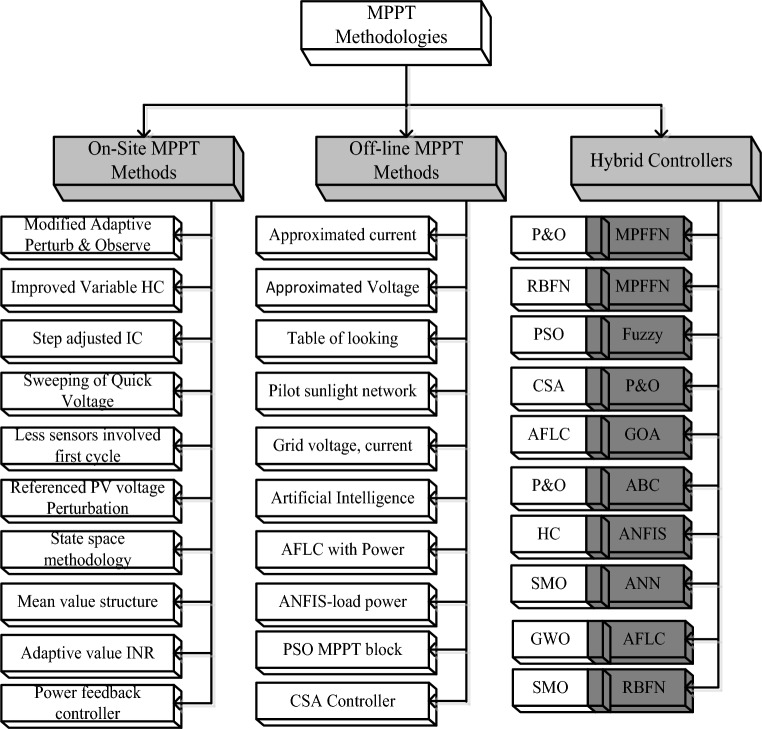
Detailed differentiation of power-point identifiers for different sunlight intensity value.

The sunlight system cells are integrated to develop the module to meet the local load demands. The sunlight system power is increased by interlinking the overall PV modules. The PV networks are implemented by applying the various concepts that are thin-film crystalline with glass coated, plus monocrystalline silicon layers. Here, the monocrystalline methodology is selected for implementing the sunlight system. Sunlight systems are produced by utilizing the diverse types of PV cell circuits. In Ref.^7^, the researchers studied the 1-diode circuit, 2-diode sunlight system, plus 3-diode circuit-dependent solar system in terms of availability of power, fill factor of the sunlight system, plus effective functioning. From this study, the authors decided that the 3-diode network is mostly preferable for the effective performance of the sunlight network. The sunlight available voltage is nonlinear. To achieve the linear voltage, the MPPT controllers are interlinked with the wind/fuel stack/solar power network. The present preference of these controllers is illustrated in Fig. 1, and the overall demonstration of MPPT methods for renewable power networks is discussed in Fig. 2.

From Fig. 2, the working MPPT methodologies are classified as on-site, hybrid, plus conventional methodologies. From the past recently existing MPPT methodologies, the adjusted step Perturb & Observe is applied to the sunlight-based battery charging network for running the entire wind/solar/battery network at quick changes of sunlight temperature values^8^. In this technology, the rapid variation of the power curve is selected to identify the actual working point of the hybrid power grid. If the power curve value is positive then the operation of the controller continues similarly. Otherwise, the controller tries to move in a reverse way. The merits of this controller block are easy to learn, more steady state powers, low-level current rating sensors are applied, plus rapid controller response. But this controller provides highly distorted sunlight power, plus more heating losses. So, the improved IC concept is utilized in the solar/battery power supply network. In this load conductance value is adjusted to achieve the peak voltage of the sunlight network. The load conductance value is equated to the source conductance by introducing the maximum power distribution concept. This IC methodology's advantages are quick MPP finding, medium accuracy, plus high reliability. However, this controller development cost is higher when associated with the P&O^9^.

The sliding methodology is interfaced with the wind/solar power production system for axial MPP identification of the wind energy network. This controller is more applicable for rooftop-installed sunlight systems because it extracts the peak voltage from time to time in a day thereby the operating efficiency of the wind system is improved. Here, the sunlight system surface conditions are developed for tracking the operating point of solar PV in a particular region. The modified sliding methodology is designed in Ref.^10^ for equal voltage sharing of the parallelly placed batteries charging. Here, all the electric vehicle interfaced battery charging stages are optimized by applying the sliding network. The steps utilized on the battery state of the charge curve are varied to improve the lifetime of the battery's working condition. The merits of this network are fast adaptability with the rapid changes of the sunlight intensity conditions, plus applicability for all types of large-scale power systems. However, this controller's demerits are more noise, plus creates high vibrations in the entire network.

**Figure 3:**
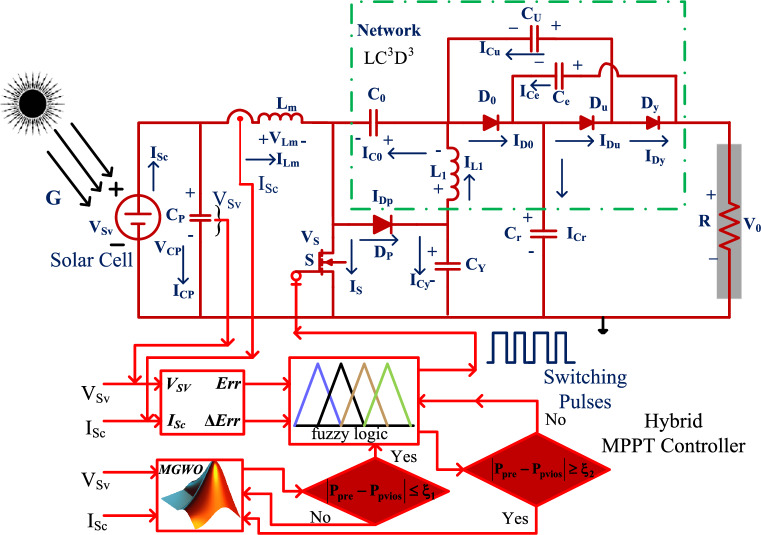
Wide voltage supply DC-DC power converter circuit with MGWM-AFLC.

The adjusted resistance methodology is selected in the sunlight battery-operated vehicles to limit the drawbacks of slider technology^11^. This variable resistance block utilizes the solar V-I curve slope value for identifying the actual functioning point of the hybrid power production network. This is very effective for the shaded PV modules. However, the nonstop variation of shaded conditions, these conventional controllers may not give the proper position of the solar MPP. In this work, the MGWM-AFLC is developed for the sunlight-based standalone power system. This controller captures the all-sunlight parameters which are solar current, PV power, plus sunlight voltage. The introduced controller working flow is illustrated in Fig. 3. From Fig. 3, at the initial stage of the power point tracking controller, the grey wolf optimization block tries to shift the functioning point of the solar system from any local place region to the actual global MPP place. The features of this proposed MPPT technology are more adaptability for any irradiation condition, fast MPP identifying speed, less dependence on the sunlight working principle, plus low complexity. Also, this method does not need a high number of sensors thereby optimizing the development cost of the entire sunlight power network.

The sunlight network voltage is varied from high level to low level based on the availability of sunlight insolation’s. To maintain the balanced voltage at the load, there are several categories of power transmission converters are reviewed in the article^12^ for optimizing the consumer load currents. The quasi-category power converter topology is useful for equal voltage distribution to the smart grid network. This converter circuit involves four capacitors, plus three inductors for increasing the voltage conversion ratio of the sunlight network. The driver circuit for this converter is applied to supply the pulses to the two semiconductor devices. The major issues of this converter are high complexity in internal structure, more space for converter driver installation, more cost, plus high-power losses due to the more passive components involved in the converter design. The quadratic Z-source power transmission converter is selected in the wind/solar system to overcome the drawbacks of a quasi-converter. Here, a Z-source circuit is useful for balancing the load power irrespective of changes in sunlight temperature values. Also, this converter performs buck operation and boost operation to providea greater range of load voltages^13^.

In the wind/battery network, the wide voltage supply power transmission converter technology is selected for charging the battery with uniform voltage. There are five power switches are involved in this circuit to optimize the current stress on the diodes. In Ref.^14^, the 2-level, and 3-level DC-DC converters are explained for the microgrid renewable energy network. The major features of this converter structure are optimal filter size, reduced voltage strain of the switch, more reliability, more suitable dynamic response, plus more suitable for high input voltage rating applications. However, these power converters' built-up costs are higher. Also, the size of the overall network is increased. In this work, a new wide source voltage power converter circuit is proposed to reduce the size of the sunlight power system thereby reducing the power consumption loss of the passive components. These converter-switching states are completely depending on the genetic optimization of AFLC as illustrated in Fig. 3.

## Development of sunlight 3-diodes PV system

**Figure 4:**
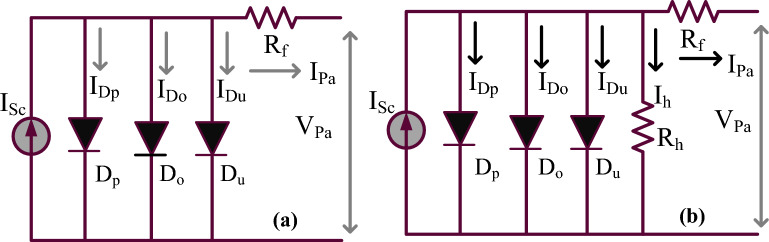
3-diode sunlight power production network, (**a**) Shunt resistive element neglected, plus (**b**) Utilizing shunt element.

The improvement of PV network efficiency is a quite difficult task because of its higher manufacturing cost, plus low accuracy levels. In Ref.^15^, there are multiple categories of sunlight PV cells which are 1-diode circuits, 2-diode-based PV circuits, plus 3-diode solar cells. The 1-diode category of the sunlight system is implemented by neglecting the junction charge recombination, plus diodes reverse leakage currents. Due to the neglect of these variables, the 1-diode network circuit design is very simple, plus simple in PV module structure. Also, this PV network used five parameters for PV string modeling which are illustrated as shunt resistive component (R_h_), selected diode factor at ideal state (ϒ), series resistive component (R_f_), sunlight current (I_Sc_), plus saturated availed diode currents (I_0P_). The limitations of a 1-diode circuit are eliminated by focusing on the 2-diode network. Here, the junction charge transformation is taken into consideration for manufacturing the sunlight module. In this one more diode is reconfigured in the 1-diode model for generating accurate output power of the solar network^16^. The 2-diodes circuit also faces the issue of leakage currents appearing across the diodes. So, in this work, a 3-diode PV module circuit is considered by selecting the diode leakage currents, plus the charge transfer effect, and its related sunlight system is represented in Fig. 4a and b. The design constraints of solar PV networksare discussed in Table 1.

**Table 1:** Selected constraints of 3-diode solar power production station.

Variables	Values
At the shaded condition of sunlight system voltage (V_Pa_)	30.11 V
At the shaded condition of sunlight system current (I_Pa_)	8.4201 amps
At the shaded condition of sunlight system power (P_Pa_)	255.007Watts
The sunlight system circuit is opened then the PV voltage (V_OCSV_)	38.221 V
Sunlight network shorted then the PV current (I_SCSC_)	8.7981 amps
Utility voltage at different thermal values	− 0.02998%/deg.c
The thermal value of sunlight current at a shaded condition	0.048876%/deg.c
Various diode factors at the ideal condition of the system (ϒ_p_, ϒ_o_, plus ϒ_u_)	0.91, 0.9, 0.89
Evaluated sunlight network series resistive component (R_f_)	0.4876 Ώ
Evaluated sunlight network series resistive component (R_h_)	350.325 Ώ
Saturation current quantities of diodes (I_op-p_, I_op-o_, I_op-u_)	1.39*10^−10^ amps

1 $${\text{I}}_{\text{Pa}} = {\text{I}}_{\text{Sc}} - {{\text{I}}}_{{\text{D}}_{\text{p}}} - {\text{I}}_{{\text{D}}_{\text{o}}} - {\text{I}}_{{\text{D}}_{\text{u}}}$$

2 $${\text{I}}_{\text{Pa}}={\text{I}}_{\text{Sc}}-{\text{I}}_{\text{op}\_\text{p}}\left({\text{e}}^{\frac{\text{q}\left({\text{V}}_{\text{Pa}}+{\text{I}}_{\text{Pa}}\ast {\text{R}}_{\text{f}}\right)}{{\Upsilon }_{\text{p}}\ast \text{KT}}}-1\right)-{\text{I}}_{\text{op}\_0}\left({\text{e}}^{\frac{\text{q}\left({\text{V}}_{\text{Pa}}+{\text{I}}_{\text{Pa}}\ast {\text{R}}_{\text{f}}\right)}{{\Upsilon }_{\text{o}}\ast \text{KT}}}-1\right)- {\text{I}}_{\text{op}\_\text{u}}\left({\text{e}}^{\frac{\text{q}\left({\text{V}}_{\text{Pa}}+{\text{I}}_{\text{Pa}}\ast {\text{R}}_{\text{f}}\right)}{{\Upsilon }_{\text{u}}\ast \text{KT}}}-1\right)$$

3 $${\text{I}}_{\text{Pa}}={\text{I}}_{\text{Sc}}-{\text{I}}_{\text{Dp}}-{\text{I}}_{\text{Do}}-{\text{I}}_{\text{Du}}-{\text{I}}_{\text{h}}$$

4 $${\text{I}}_{\text{Sc}}={\text{I}}_{\text{Pa}}-{\text{I}}_{\text{op}\_\text{p}}\left({\text{e}}^{\frac{\text{q}\left({\text{V}}_{\text{Pa}}+{\text{I}}_{\text{Pa}}\ast {\text{R}}_{\text{f}}\right)}{{\Upsilon }_{\text{p}}\ast \text{KT}}}-1\right)-{\text{I}}_{\text{op}\_0}\left({\text{e}}^{\frac{\text{q}\left({\text{V}}_{\text{Pa}}+{\text{I}}_{\text{Pa}}\ast {\text{R}}_{\text{f}}\right)}{{\Upsilon }_{\text{o}}\ast \text{KT}}}-1\right)-{\text{I}}_{\text{ptl}}$$

5 $${{\text{I}}}_{{\textrm{Ptl}}}={{\text{I}}}_{{\textrm{op}}\_{\text{u}}}\left({{\text{e}}}^{\frac{{{\text{q}}\left({{\text{V}}}_{{\textrm{Pa}}}+{{\text{I}}}_{{\textrm{Pa}}}\ast {{\text{R}}}_{{\textrm{f}}}\right)}}{{{\Upsilon }_{\text{u}}\ast \text{KT}}}}-1\right)+{\frac{{{\text{V}}_{\textrm{Pa}}+{\text{I}}_{\textrm{Pa}}{\text{R}}_{\textrm{f}}}}{{{\text{R}}_{\textrm{h}}}}}$$

6 $${\text{I}}_{\text{op}\_\text{p}}={\text{I}}_{\text{op}\_\text{o}}={\text{I}}_{\text{op}\_\text{u}}={\text{I}}_{\text{ond}}\ast (\frac{\text{T}}{{\text{T}}_{\text{ond}}}{)}^{3}{\text{ e}}^{\frac{\text{q}\ast \text{Eg}}{\text{n}\ast \text{k}}\left(\frac{1}{{\text{T}}_{\text{onc}}}-\frac{1}{\text{T}}\right)}$$

7 $${\text{I}}_{\text{ond}}={\text{I}}_{\text{on}\_\text{p}}={\text{I}}_{\text{on}\_\text{u}}={\text{I}}_{\text{on}\_\text{o}}=\frac{{\text{I}}_{\text{Sci}\_\text{n}}}{{\text{e}}^{\left(\frac{{\text{V}}_{\text{oc}\_\text{n}}}{\Upsilon \ast {\text{V}}_{\ast \text{Tn}}}\right)}}$$

### Shading effect on sunlight-dependent PV modules

**Figure 5:**
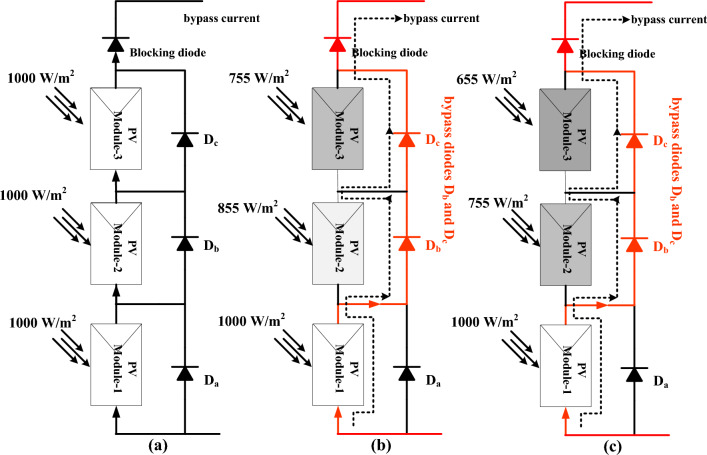
(**a**) Constant sunlight isolation, (**b**) shading pattern-1, plus (**c**) Shaded pattern-2 condition.

Now, all the categories of sunlight systems are located on the top of the buildings and shadow-free places. However, due to the atmospheric cloudy circumstances, there is a shaded effect on the sunlight modules^17^. As a result, the selected sunlight system is unable to deliver the peak supply voltage. In this situation, the power curves of the solar system consist of more than two MPPs, and there is a possibility that the solar system can function at any one local MPP place. So, the power production from the solar is reduced. Here, there are “3” cases of partially shaded values utilized for testing the sunlight network throughout the day, and the pictorial representation of shaded modules is discussed in Fig. 5. From Fig. 5a, the three modules captured solar insolation values are equal which is identified as 1000W/m^2^. The obtained sunlight power, solar voltage, plus sunlight production currents under uniform insolation conditions are 762W, 90.30 V, plus 8.43A. At first shaded pattern, the observed sunlight insolation valuesfor the three PV modules are 1000W/m^2^, 855W/m^2^, plus 755W/m^2^ as discussed in Fig. 5b. Here, at a constant insolation value of 1000W/m^2^, the antiparallel connected bypass diodes (D_a_, D_b_, plus D_c_) are in a normal state.

At shading pattern-1, the module-1 receives 1000W/m^2^ and its related device D_a_ is in ideal condition. The remaining modulescaptures 855W/m^2^, plus 755W/m^2^ irradiations and its related devices D_b_, plus D_c_start functioning in forward bias condition. Similarly, in the 1^st^shading pattern, the sunlight network modules work in the same fashion. The availed sunlight voltage, solar power, plus currents under 2nd solar shaded condition are 89.42 V, 606.267W, plus 6.78A as given in Fig. 5c. Finally, the captured sunlight irradiations at 2nd shading are 1000W/m^2^, 755W/m^2^, plus 655W/m^2^ and its associated sunlight system power, PV current, plus system voltages are 543.12W, 6.371A, and 86.452 V respectively.The sunlight system produced mismatch variance power loss, plus misleading divergence power losses are 155.733W, plus 63.147W.

## Design of different MPPT controllers for sunlight system

The sunlight network shadingand rapid variation of atmospheric temperature conditions create a big impact on the solar system's operating efficiency. Also, the starting installation price of the PV network is higher because of low-quality manufacturing technologies^18^. Due to these issues, the PV networks have to capture the entire sunlight intensity. From the literature study, the power point tracing methodologies are machine learning, soft computing, deep learning, plus artificial neural controllers. In this manuscript, the GWM along with AFLC is analyzed for the uniform, plus shaded conditions of the sunlight values. The proposed hybrid methodology is investigated by considering the other algorithms which are Adaptive Multilayer Perceptron Artificial Neural Controller-Proportional and Integral (AMPANC-PI), Improved Radial Basis Function Artificial Controller-PI (IRBFAC-PI), plus Improved Genetic Optimization-Perturb & Observe (IGO with P&O). The MPPT methodologies are examined by utilizing the variables that trace MPP speed, development complexity, plus how many dependencies on PV network modeling.

### AMPANC-PI power point identifying controller

**Figure 6:**
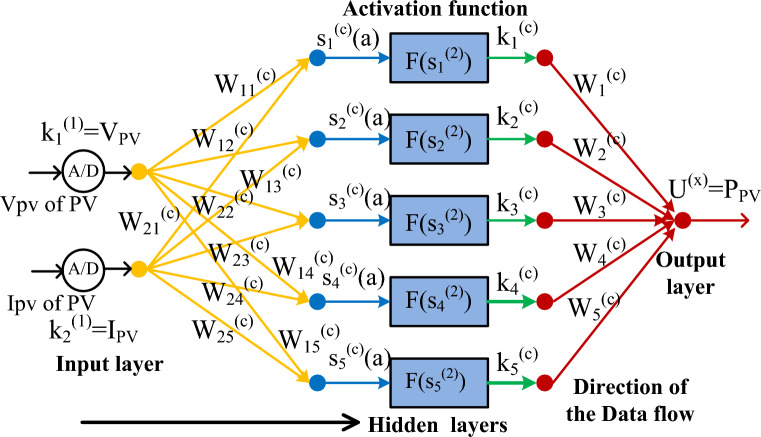
Improved perceptron multilayer artificial neural controller, Ref.^19^.

Now, the MPPT study says that the conventional controller is less utilizable forrapid variation of atmospheric values. Especially, the P&O, plus INR methodologies suffer from distorted converter supply voltages, plus continuous variations in the solar MPP region. Also, these methods' operational costsare higher when associated with artificial intelligence controllers. In the manuscript^19^, a multilayer perceptron artificial intelligence methodology is selected for the wind/solar power production network to improve the functioning stability of the entire system. This concept is designed based on the brain functioning of human beings. In our human brain, thousands of neurons are interconnected and work by exchanging their searching data. The functioning of the perceptron neural method is illustrated in Fig. 6. From Fig. 6, in the perceptron network, the first layer neurons are initialized by selecting the sunlight voltage, plus PV current. Here, the input layer nodes in the AMPANC are “2”. The second layer collects the source layer data for adjusting the solar network output voltage from one place of the V-I curve to another region. The available neurons in the middle layer are “7”, and the weight is increased based on the linear activation function. Finally, the output node produces the error sunlight voltage which is sent to the PI-based pulse generation block to achieve the required duty for the proposed converter.

8 $${\text{S}}_{\text{h}}^{(2)}(\text{a})={\sum }_{\text{r}=1}^{2}{\text{W}}_{\text{hr}}^{(2)}\ast {\text{Q}}_{\text{r}}^{1};\text{r}=\text{1,2},3......,7$$

9 $${\text{Q}}_{\text{h}}^{(2)}(\text{a})=\text{f}\left({\text{S}}_{\text{h}}^{(2)}(\text{a})\right)$$

10 $${\text{L}}^{3}(\text{a})={\sum }_{\text{r}=1}^{5}{\text{W}}_{\text{r}}^{(3)}\ast {\text{L}}_{\text{r}}^{(2)}$$

11 $${\text{W}}_{\text{hr}}^{(2)}={\text{W}}_{\text{hr}}^{(2)}+\Delta {\text{W}}_{\text{hr}}$$

12 $${\text{W}}_{\text{h}}^{(3)}={\text{W}}_{\text{h}3}^{(3)}+\Delta {\text{W}}_{\text{r}}$$

13 $$\Delta {\text{W}}_{\text{hr}}=\text{A}\ast \frac{\partial \text{e}}{\partial {\text{W}}_{\text{hr}}^{\left(2\right)}}, \&\Delta {\text{W}}_{\text{r}}=\text{A}\ast \frac{\partial \text{e}}{\partial {\text{W}}_{\text{r}}^{(3)}}$$

14 $$\text{e}=\frac{1}{2}{\left({\text{P}}_{{\text{desired}}_{\text{PV}}}-{{\text{P}}_{\text{PV}}}^{\left(3\right)}\right)}^{2}$$

where the variables k, W, S, plus U are source layer nodes, the weight of each node, the second layer output signal, plus the final layer output signal. Here, the Q, L, plus e are the net values of the 1st, 2nd, plus 3rd layers, and the error power signal of the final layer.

### IRBFAC-PI power point identifying controller

**Figure 7:**
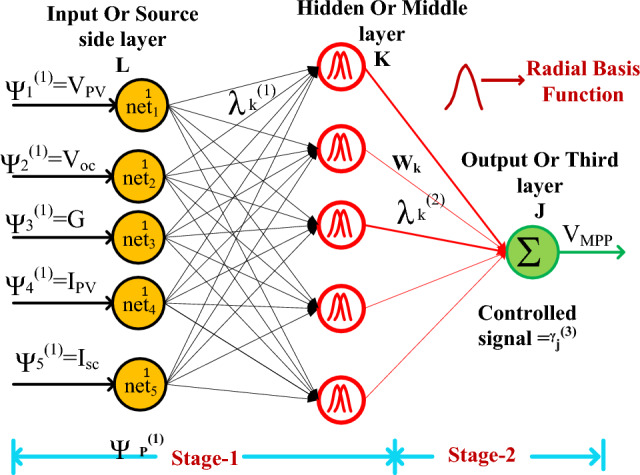
IRBFAC-PI power point identifying controller for sunlight network.

The artificial intelligence-based multilayer perceptron network consists of more than two layers and it needsa big data set to obtain the suitable objective value. Due to the excess data training, the convergence time of this controller takes more time^20^. Also, the perceptron network takes only either “0” or “1” because of the hard limit transfer function and it only works for linearly separable vectors. So, the adjusted step-modified RBFAC is integrated with the solar water pumping system to maintain the constant induction motor speed. This method takes only “3” layers and it needs very low training data for obtaining the peak power point region. This method tries to linearize the nonlinear performance of the sunlight system, and its implementation cost and complexity are low when equated to the perceptron network. The working of RBFAC for the shaded sunlight network is mentioned in Fig. 7. From Fig. 7, the variables ψ, ʎ, L, K, plus J are the source layer inputs, the net value of the source layer, plus 1st, 2nd, plus 3rd layer numbers.

15 $${\upbeta }_{\text{L}}^{1}\left(\text{q}\right)={\text{f}}_{\text{L}}^{1}{\text{net}}_{\text{L}}^{1}\left(\text{q}\right)={\text{net}}_{\text{l}}^{1}{\text{(q)}};{\text{ q}}=\text{1,2}\dots ..\text{z}$$

16 $${\text{net}}_{\text{K}}^{2}(\text{q})=-(\text{A}-{\text{m}}_{\text{K}}{)}^{\text{T}}{\sum }_{\text{k}}\left(\text{A}-{\text{m}}_{\text{k}}\right);\text{q}=1.2.3...,\text{z}$$

17 $${{\rotatebox{180}{y}}_{\text{k}}^{2}}(\text{q})={{\text{f}}_{\text{k}}^{2}}{{\text{net}}_{\text{k}}^{2}}(\text{q})={{\text{net}}_{\text{k}}^{2}}{\text{(q)}}; {\text{q}}=1.2..,\text{z}$$

18 $${{\text{net}}_{\text{J}}^{3}}={{\sum }_{\text{k}}}{{\text{W}}_{\text{k}}}\ast {{\rotatebox{180}{y}}_{\text{j}}^{2}}{\text{(q); q}}=1.2.3....,\text{z}$$

19 $${{\rotatebox{180}{y}}_{j}^{3}}(\text{q})={{\text{f}}_{\text{j}}^{3}}{{\text{net}}_{\text{j}}^{3}}(\text{q})={{\text{net}}_{\text{j}}^{3}}{\text{(q); q}}=1.2...,\text{z}$$

20 $${\text{error value}}={{\sum }_{\text{q}=1}^{\uppsi }}{\frac{1}{2}}\left({{\text{V}}_{\text{PV}-\text{ref}}}-{{\text{V}}_{\text{MPP}}}\right)$$

### IGO with P&O power point identifying controller

Till now, we have seen many conventional, machine-learning, and artificial intelligence controllers. From the literature data, these methods take much more time to find the required target value^21^. So, the genetic concept is included in the conventional controller for enhancing the MPP finding speed. In this controller, the P&O method takes the working point of the sunlight system from the general place to the peak power place, and it takes a very low iteration number thereby the genetic optimization concept helps the sunlight system for fast system response. Later, the genetic method optimizes the distortions of the sunlight production power. The evaluation of suitable duty value for the introduced converter is mentioned in Eqs. (21), and (22). From Eq. (21), the functioning pointy of the sunlight network for away from the right-hand side of the MPP then the duty is adjusted positively. Otherwise, the duty is optimized by taking the variable step value as mentioned in Eq. (23).

21 $$\text{D}\left(\text{h}\right)=\text{D}\left(\text{h}-1\right)+\text{\pounds }\ast \left(\frac{\text{P}(\text{h})-\text{P}(\text{h}-1)}{\text{V}(\text{h})-\text{V}(\text{h}-1)}\right)$$

22 $$\text{D}\left(\text{h}\right)=\text{D}\left(\text{h}-1\right)-\text{\pounds }\ast \left(\frac{\text{P}(\text{h})-\text{P}(\text{h}-1)}{\text{V}(\text{h})-\text{V}(\text{h}-1)}\right)$$

23 $$\text{Step Value }=\text{\pounds }\ast \left(\frac{\text{P}(\text{h})-\text{P}(\text{h}-1)}{\text{P}(\text{h})-\text{P}(\text{h}-1)}\right)$$

where P(h-1), D(h-1), £, P(h) plus D(h) are the stored sunlight powers, duty signal of the converter, plus step value of the controller, instantaneous powers, and duty values.

### MGWM-AFLC power point identifying controller

**Figure 8:**
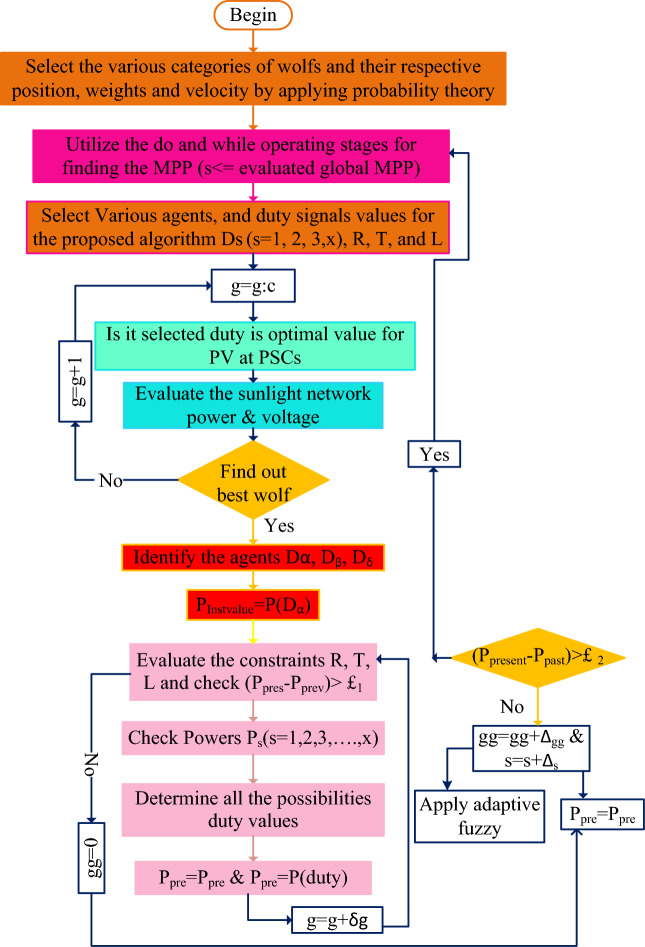
Proposed GWM for AFLC for sunlight system application.

The above studied power point finding controllers face the issue of more training time, plus takes high development cost. In this work, the modified GWM is selected for the AFLC membership values selection. From the literature review, the basic fuzzy controller’s accuracy is evaluated based on the membership functions^22^. Also, it has the problems of grounding, utilizing complex problems, needed testing equipment, and forming. So, the easiest technique for identifying the proper fuzzy membership is the grey wolf methodology. Here, the grey wolf agents are initialized with various weights by selecting the probability theory. At the beginning stage, the wolf runs in multiple ways. After certain iterations, the wolves try to run in a unique way to evaluate the optimal solution for the particular issue. Once, the wolf goes to the actual working point of the sunlight system, the fuzzy collects the wolf's running information and optimizes the oscillations of the sunlight power. The functioning of this controller is indicated in Fig. 8. From Fig. 8, there are two formulas designed for running the two controllers alternatively which are Eq. (24) is applied once the grey wolf searching mechanism is completed for finding the required objective function then the entire network operation tries to move on the fuzzy block. Otherwise, the Eq. (25) is selected for doing the reverse operation of the controller.

24 $$\left|{\text{Power}}_{\text{present}}-{\text{Power}}_{\text{previous}}\right|\le {\text{\pounds }}_{1}$$

25 $$\left|{\text{Power}\_\text{PV}}_{\text{present}}-{\text{Power}\_\text{PV}}_{\text{previous}}\right|\ge {\text{\pounds }}_{2}$$

where the terminologies are present PV power, plus already availed powers are equated to find out the peak power of the solar system. Finally, £ is the applied condition limiting factor.

## Design of wide power rating high voltage DC-DC boost converter

The present isolated networks need more power electronics components, andthey work with more duty value^23^. So, the entire system may not generate the accurate load power. Also, these converters utilize an excessive number of power switches, plus power diodes. As a result, the converter utilization factor is very low. In this work, a wide power rating, single switch-based DC-DC converter circuit is introduced for enhancing the sunlight system voltage profile thereby supplying the peak voltage to the consumer load. The utilized power switches and diodes are named S, D_P_, D_0_, D_U_, plus D_Y,_ and their related currents, plus supplied voltages are I_S_, I_DP_, I_D0_, I_DU_, I_DY_, V_S_, V_DP_, V_D0_, V_DU_, plus V_DY_ respectively. Here, there are “3” approximations utilized for evaluating the voltage conversion ratio of the sunlight network which are 1st one is that the utilized passive elements' internal resistive nature is neglected, and 2nd is all the power devices are in ideal mode. Finally, the 3rd assumption is supply power is quite equal to consumer power.

### 1st functioning state of the power switch

**Figure 9:**
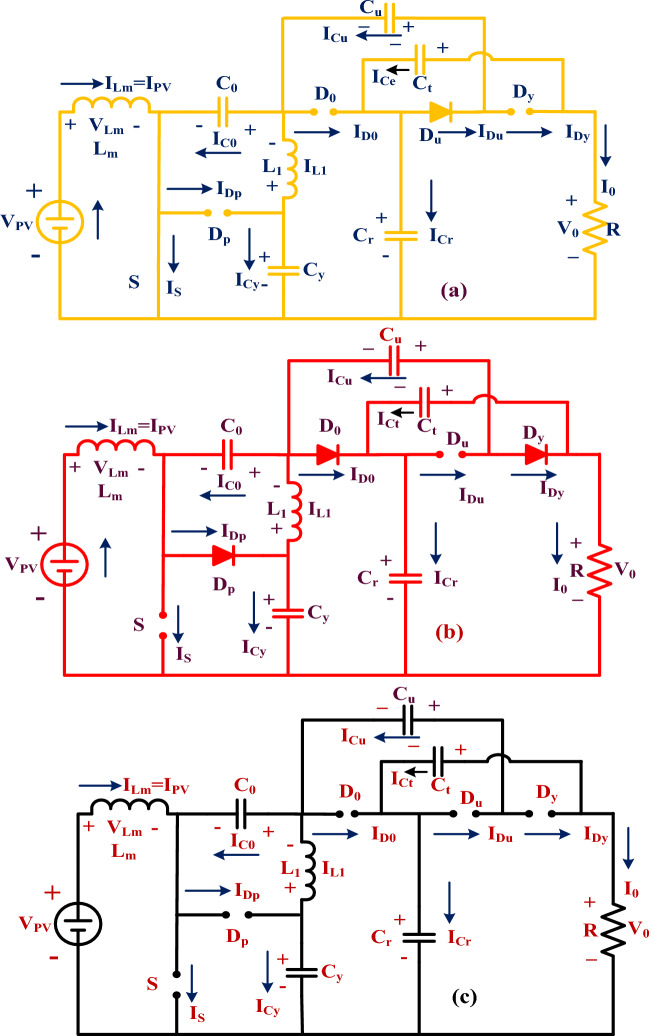
(**a**) Power switch functioning, (**b**) The power switch stops responding, plus (**c**) all power devices stop responding tothe supply.

In this functioning mode of the power circuit, the source voltage utilized to the gate is greater. Due to this more gate supply voltages, the power conversion circuits operate in uniform power production conditions as well as distorted power supply mode. The power transfer circuit function stages are mentioned in Table 2. Here, based on Table 2, the power switch (S) starts working with the help of the gate signal, plus the power diodes D_P_, D_0_, plus D_Y_ are moving into the cutoff region as given in Fig. 9a. The source inductors L_m_, plus L_1_ start linearly consuming the energy. Similarly, the capacitive elements C_p_, plus C_0_ try to consume the voltage, and C_u_, C_y_ plus C_r_ supplies the voltage to the consumers through the diodes. The voltages of the power transfer circuit are mentioned in Eqs. (26), and (27).

**Table 2:** Power transfer circuit functioning stages at diverse sunlight values.

Elements	1st mode (continuous & fluctuate)	2nd (continuous & fluctuate)	Fluctuate mode
S	Operating state	Stops responding	Stops responding
D_P_	Stops responding	Operating state	Stops responding
D_0_	Stops responding	Operating state	Stops responding
D_u_	Operating state	Operating state	Stops responding
D_y_	Stops responding	Operating state	Stops responding

26 $$\left\{\begin{array}{l}{\text{V}}_{\text{Lm}}={\text{V}}_{\text{Pa}}\\ {\text{V}}_{\text{L}1}={\text{V}}_{\text{Cy}\_\text{dirg}}-{\text{V}}_{\text{C}0\_\text{cltg}}\end{array}\right.$$

27 $$\left\{\begin{array}{l}{\text{I}}_{\text{C}0\_\text{cltg}}={\text{I}}_{\text{Switch}}-{\text{I}}_{\text{Lm}}\\ {\text{I}}_{\text{C}0\_\text{cltg}}-{\text{I}}_{\text{Cu}\_\text{cltg}}={\text{I}}_{\text{L}1}=-{\text{I}}_{\text{Cy}\_\text{dirg}}\\ {\text{I}}_{\text{Cr}\_\text{dirg}}={\text{I}}_{\text{Cu}\_\text{cltg}}+{\text{I}}_{\text{Cr}\_\text{dirg}}=-{\text{I}}_{0}\end{array}\right.$$

### 2nd & 3rd functioning state of the power switch

**Figure 10:**
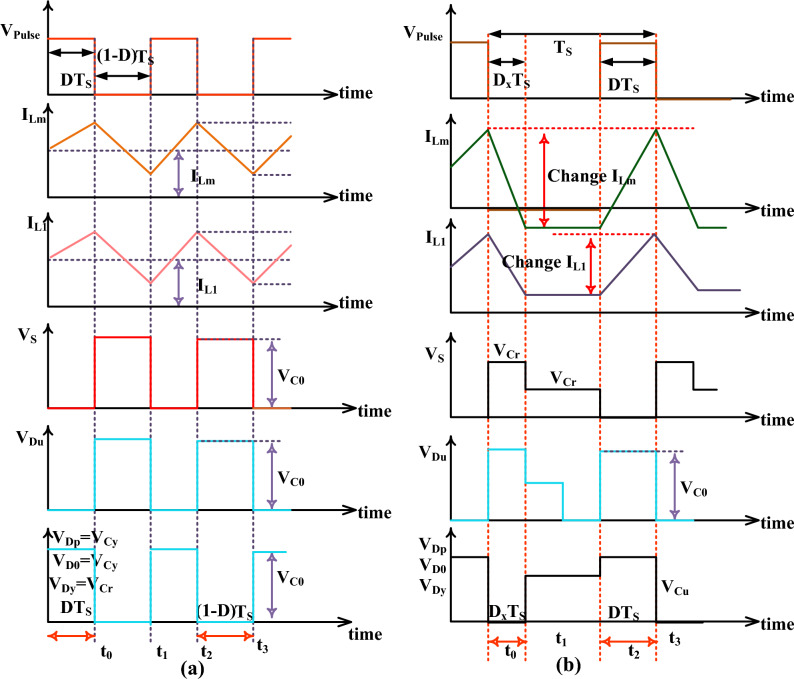
(**a**) Power switch working mode, plus (**b**) stop functioning the power switch.

The energy storage and discharge capacitors are identified as I_Cp-cltg_, I_C0-cltg_I_Cu-cltg_, I_Cy-cltg_, I_Cr-cltg_, I_Cp-dirg_, I_C0-dirg_, I_Cu-dirg_, I_Cy-dirg_, plus I_Cr-dirg_, and the related capacitive voltages are V_Cp-cltg_, V_C0-cltg_V_Cu-cltg_, V_Cy-cltg_, V_Cr-cltg_, V_Cp-dirg_, V_C0-dirg_, V_Cu-dirg_, V_Cy-dirg_, plus V_Cr-dirg_ respectively. In this second stage, the power switch moves in blocking mode, and diodes D_P_, D_0_, plus D_Y_ supply the current to the resistor (R). Here, the capacitive components C_u_, C_y_ plus C_r_ deliver the initial stored power supply, plus C_p_, plus C_0_ capture the load voltage as discussed in Fig. 9b. From Fig. 9c, the source voltage transformation completely depends on the inductors L_m_, plus L_1_. Here, the inductors supply voltages and currents are named V_Lm-cltg_, V_L1-cltg_, V_Lm-dirg_, V_L1-dirg_, I_Lm-cltg_, I_L1-cltg_, I_Lm-dirg_, plus I_L1-dirg_. The voltage conversion, plus the current transformation of the converter circuit is discussed in Eqs. (28), and (29). In the final working mode of the converter circuit, all the power devices stop responding to the solar power source. Here, the mean value of the inductive currents is “0”, and voltages are equal to “0”. The power converter functioning waveforms are explained in Fig. 10a, plus Fig. 10b.

28 $$\left\{\begin{array}{l}{\text{V}}_{\text{Lm}}={\text{V}}_{\text{Pa}}-{\text{V}}_{\text{Cy}\_\text{cltg}}\\ {\text{V}}_{\text{L}1}={\text{V}}_{\text{Cy}\_\text{cltg}}-{\text{V}}_{\text{Cr}\_\text{cltg}}\end{array}\right.$$

29 $$\left\{\begin{array}{l}{\text{I}}_{\text{C}0\_\text{dirg}}={\text{I}}_{\text{Dp}}-{\text{I}}_{\text{Lm}}\\ {\text{I}}_{\text{Cu}\_\text{dirg}}={\text{I}}_{\text{D}0}+{\text{I}}_{\text{C}0\_\text{dirg}}-{\text{I}}_{\text{L}1}\\ {\text{I}}_{\text{Cy}\_\text{cltg}}={\text{I}}_{\text{L}1}-{\text{I}}_{\text{Dp}}\\ {\text{I}}_{\text{Ct}\_\text{cltg}}={\text{I}}_{\text{Cr}\_\text{cltg}}-{\text{I}}_{\text{D}0}\end{array}\right.$$

30 $${\text{V}}_{\text{Lm}\_\text{Minimum}}={\text{V}}_{\text{L}1\_\text{Minimum}}=0$$

31 $${\text{I}}_{\text{Lm}\_\text{Minimum}}+{\text{I}}_{\text{L}1\_\text{Minimum}}=0$$

### Converter steady state and comprehensive analysis

**Figure 11:**
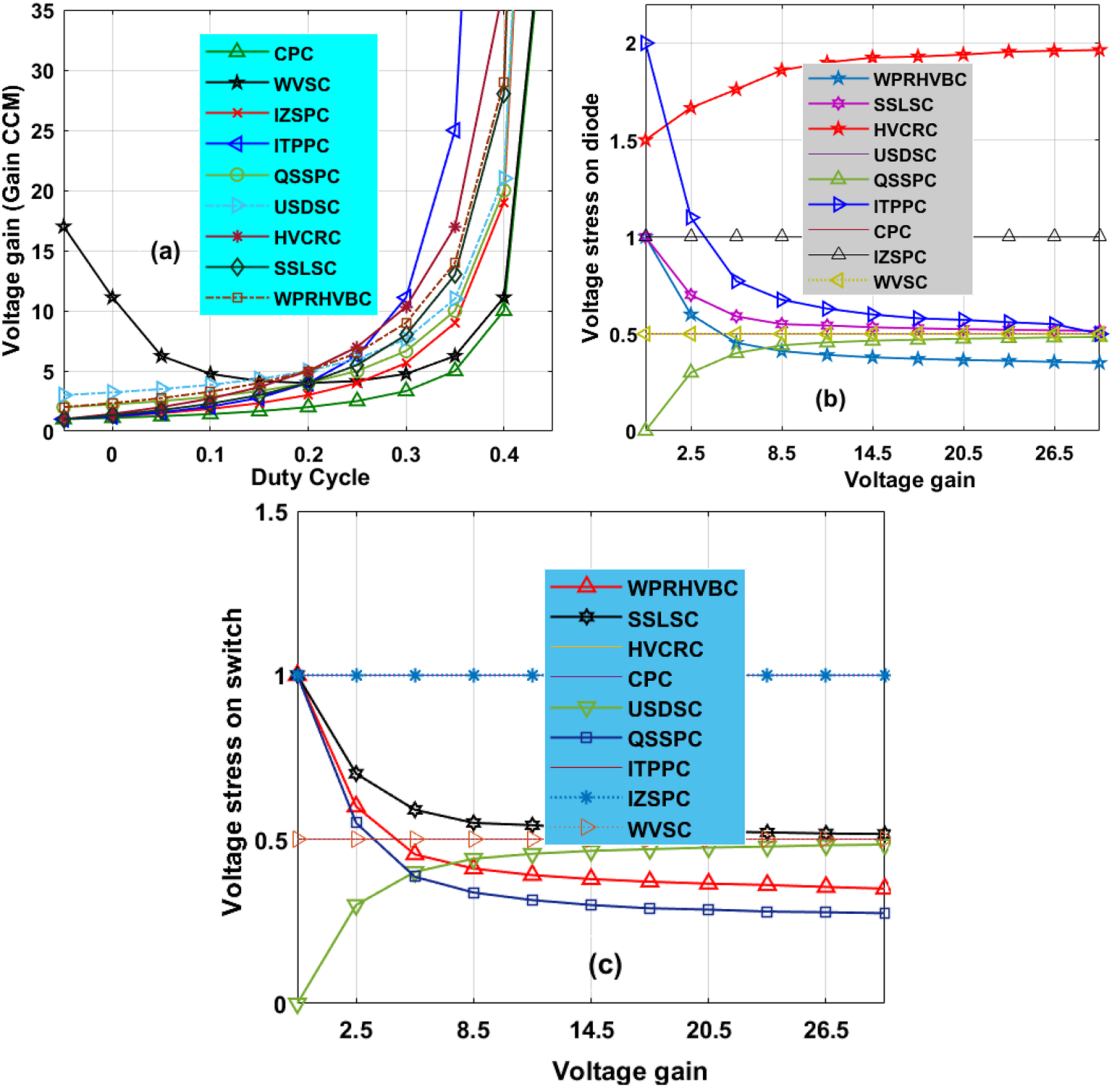
(**a**) Change in VCR by varying the duty, (**b**) Change in voltage of diode with VCR, plus (**c**) Change in voltage of switch with VCR.

From Fig. 10a, the converter's continuous functioning and fluctuated function stages are utilized for the investigation of the steady-state response of the power transfer circuit. The voltage conversion ratio of the circuit is evaluated by applying Kirchhoff’s voltage rule. From Fig. 10b, the power switch voltage rises from origin to V_C0_ which is mentioned in Eqs. (32) to (40). From Eq. (40), the current appeared across the inductor is evaluated by approximating the source power (P_Pa_ = V_Pa_* I_Pa_) with the consumer power (P_P0_ = V_P0_* I_P0_). Here, the introduced converter comprehensive study has been made along with the other power transfer circuits as mentioned in Table 3. The variables CCM defines continuous conduction mode and VCR is illustrated as voltage conversion ratio. Finally, the variable DCM defines the discontinuous mode of the power transfer circuit. The changes of VCR with duty, voltage of the diode with VCR, plus voltage of the switch with VCR are illustrated in Fig. 11a–c.

**Table 3:** Analysis of power conversion circuits under fast variation of sunlight intensity conditions.

Type of circuit	Gain of circuit	Used elements	Need ground	Needed L, plus C	Nature of I	Stress of MOSFET	Stress of diode
CPC^24^	$\frac{1}{1-\text{Duty}}$	1-S, 1-D	Not require	1-L, 1-C	Uniform	1	1
WVSC^25^	$\frac{1+2\text{Duty}}{1-\text{Duty}}$	1-S, 3-D	Require	3-L, 5-C	Uniform	$\frac{{\text{VCR}}_{\text{CCM}}+2}{3{\text{VCR}}_{\text{CCM}}}$	$\frac{{\text{VCR}}_{\text{CCM}}+2}{3{\text{VCR}}_{\text{CCM}}}$
IZSPC^26^	$\frac{1}{\text{Duty}(1-\text{Duty})}$	2-S, 3-D	Require	2-L, 2-C	Fluctuate	$\frac{1}{2}+\sqrt{\frac{1}{4}-\frac{1}{{\text{VCR}}_{\text{CCM}}}}$	$\frac{3}{2}+\sqrt{\frac{1}{4}-\frac{1}{{\text{VCR}}_{\text{CCM}}}}$
ITPPC^27^	$\frac{1+\text{Duty}}{1-\text{Duty}}$	2-S, 4-D	No	2-L, 3-C	Uniform	$\frac{1+{\text{VCR}}_{\text{CCM}}}{2\ast {\text{VCR}}_{\text{CCM}}}$	$\frac{1+{\text{VCR}}_{\text{CCM}}}{2\ast {\text{VCR}}_{\text{CCM}}}$
QSSPC^28^	$\frac{1}{(1-\text{D})(1+\text{D})}$	3-D, 1-S	Require	2-L, 2-C	Fluctuate	1	1
USDSC^29^	$\frac{3-\text{Duty}}{1-\text{Duty}}$	1-S, 4-D	No	2-L, 4-C	Fluctuate	$\frac{{\text{VCR}}_{\text{CCM}}-1}{2\ast {\text{VCR}}_{\text{CCM}}}$	$\frac{{\text{VCR}}_{\text{CCM}}-1}{2\ast {\text{VCR}}_{\text{CCM}}}$
HVCRC^30^	$\frac{1+3\text{Duty}}{1-\text{Duty}}$	2-S, 2-D	Require	3-L, 3C	Uniform	$\frac{1}{2}$	$\frac{1}{2}$
SSLSC^30^	$\frac{2}{1-\text{Duty}}$	2-S, 2-D	Require	2-L, 2-C	Fluctuate	$\frac{1}{2}$	$\frac{1}{2}$
WPRHVBC	$\frac{2+\text{D}}{1-\text{D}}$	1-S, 4-D	Require	2-L, 4-C	Uniform	$\frac{3+{\text{VCR}}_{\text{CCM}}}{4\ast {\text{VCR}}_{\text{CCM}}}$	$\frac{3+{\text{VCR}}_{\text{CCM}}}{4\ast {\text{VCR}}_{\text{CCM}}}$

32 $${\text{V}}_{\text{Co}}=\frac{\text{Duty}}{(1-\text{Duty})}{\text{V}}_{\text{Pa}}$$

33 $${\text{V}}_{\text{Cu}}={\text{V}}_{\text{Cy}}={\text{V}}_{\text{Ct}}=\frac{1}{(1-\text{Duty})}{\text{V}}_{\text{Pa}}$$

34 $${\text{V}}_{\text{Cr}}=\frac{1+\text{Duty}}{(1-\text{Duty})}\ast {\text{V}}_{\text{Pa}}$$

35 $${\text{V}}_{0}=\frac{2+\text{Duty}}{(1-\text{Duty})}{\text{V}}_{\text{Pa}}$$

36 $${\text{Gain}}_{\text{CCM}}=\frac{{\text{V}}_{0}}{{\text{V}}_{\text{Pa}}}=\frac{2+\text{D}}{(1-\text{D})}$$

37 $$\left\{\begin{array}{l}{\text{V}}_{\text{S}}={\text{V}}_{\text{D}}={\text{V}}_{\text{Pa}}\frac{1}{(1-\text{Duty})}\\ {\text{V}}_{\text{D}}={\text{V}}_{\text{Dp}}={\text{V}}_{\text{D}0}={\text{V}}_{\text{Du}}={\text{V}}_{\text{Dy}}\end{array}\right.$$

38 $${\text{V}}_{\text{S}}={\text{V}}_{\text{D}}=\frac{2+{\text{Gain}}_{\text{CCM}}}{3{\text{Gain}}_{\text{CCM}}}{\text{V}}_{0\text{utput}}$$

39 $${\text{I}}_{\text{L}1}={\text{I}}_{\text{L}2}={\text{I}}_{0}$$

40 $${\text{I}}_{\text{L}1}=\frac{2+\text{Duty}}{1-\text{Duty}}\ast {\text{I}}_{\text{out}}={\text{Gain}}_{\text{CCM}}\ast {\text{I}}_{0}$$

## Analysis of simulation results

The 3-diode circuit model sunlight power delivery system is developed by considering the MATLAB/Simulink tool. The entire sunlight network power source capability, voltage generation, plus the current flow through the load resistor are 762W, 90.30 V, plus 8.43A respectively. This triple diode concept is the most innovative technology in the solar network for producing more output voltage with good fill factor value. Also, this circuit eliminates the leakage power flow in the solar-based standalone systems and optimizes the junction charge transferring losses. The working efficiency of this circuit is quite more when related to the other solar models at constant and shading conditions of the solar PV. The shading phenomena creates a big issue in the standalone sunlight power production network and it comes from the tall building shadows, plus atmospheric cloudy conditions. Also, the dust on the sunlight PV modules causes faults in the overall solar power network.

### Analysis of MGWM-AFLC fed solar system at constant irradiation (1000 W/m^2^)

**Figure 12:**
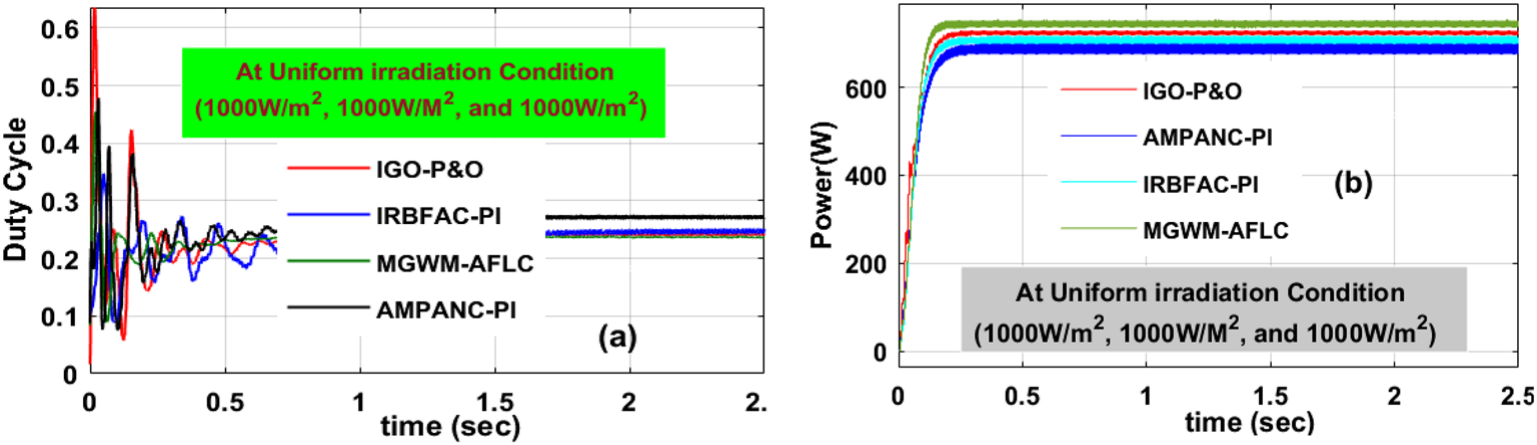
Introduced power boost DC-DC converter circuit, (**a**) duty signal, plus (**b**) DC-DC power at 1000W/m^2^ value.

In this sunlight intensity condition, the proposed system supplies more energy because all the sunlight modules capture the uniform irradiation thereby the overall network functioning efficiency is improved. In this sunlight network, the AMPANC-PI, IRBFAC-PI, IGO-P&O, plus MGWM-AFLC fed sunlight network produced converter power, voltage, duty cycle, and their related settling times are evaluated as 703.902W, 220.118 V, 0.48,0.995 s, 730.663W, 225.216 V, 0.479, 0.932 s, 751.886W, 226.99 V, 0.48, 0.599 s, 754.331W, 239.950 V, 0.42, 0.504 s respectively. From Fig. 12a, the modified grey wolf methodology evaluated the power converter functioning duty value as 0.42 which is a very low value. So, the power production of the system works very efficiently which is mentioned in Fig. 12b. Also, the MPP tracing time, plus efficiencies of the sunlight system-based AMPANC-PI, IRBFAC-PI, IGO-P&O, plus MGWM-AFLC MPPT methodologies under constant insolation value are 0.851 s, 95.012%, 0.811 s, 95.224%, 0.451 s, 96.878%, 0.477 s, plus 98.16% respectively.

### Analysis of MGWM-AFLC fed PV system at 1000 W/m^2^, 855 W/m^2^, plus 755 W/m^2^

**Figure 13:**
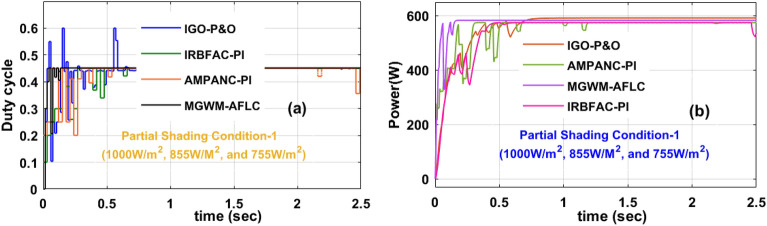
Introduced power boost DC-DC converter circuit, (**a**) duty signal, plus (**b**) DC-DC power at 1st shading of sunlight system.

In this shaded condition, the sunlight system captured insolation value is low when associated with the constant irradiation condition. So, the power production capacity of the overall network at this shading pattern is reduced. Also, power losses occur in the proposed system in the form of heat generation. There are diverse MPPT concepts utilized in this situation to improve the power transmission capacity of the network. The available converter circuit voltages, load powers, and functioning efficiencies by interfacing the AMPANC-PI, IRBFAC-PI, IGO-P&O, plusMGWM-AFLC MPPT controllers are 174.221 V, 568.551W, 94.8981%, 176.127 V, 580.544W, 94.995%, 180.22 V, 588.7714W, 95.579%, 183.225 V, 600.2141W, plus 97.921% respectively. The converter circuit voltage settling time, plus MPP tracking times by selecting the MGWM-AFLC technique are 0.521 s, plus 0.481 s which are very optimal values when related to the IGO-P&O, plus AMPANC-PI controllers. The obtained converter duty signal, plus load powers are discussed in Fig. 13(a), and b. The efficiency of the overall sunlight network is given in Table 4.

**Table 4:** Analysis of MGWM-based AFLC technique along with other MPPT techniques.

Type of Method	Voltage of the circuit	Power of the circuit	Efficiency (η)	Fluctuation in the system	Time for MPP tracking	Time of settling	Speed of MPP identifying	Accuracy of the system
Constant sunlight intensity condition of the proposed system (1000 W/m^2^)								
AMPANC-PI	220.118 V	703.902W	95.012%	More	0.851 s	0.995 s	Low	Low
IRBFAC-PI	225.216 V	730.663W	95.224%	More	0.811 s	0.932 s	Low	Low
IGO-P&O	226.99 V	751.886W	96.878%	Medium	0.451 s	0.599 s	Medium	Medium
MGWM-AFLC	239.950 V	754.331W	98.16%	Less	0.477 s	0.504 s	High	More
Utilization of 1st Shading effect on the sunlight intensity condition (1000 W/m^2^, 855 W/m^2^, plus 755 W/m^2^)								
AMPANC-PI	174.221 V	568.551W	94.8981%	More	1.032 s	1.199 s	Low	Low
IRBFAC-PI	176.127 V	580.544W	94.995%	More	0.678 s	0.853 s	Low	Low
IGO-P&O	180.22 V	588.7714W	95.579%	Medium	0.553 s	0.602 s	Medium	Medium
MGWM-AFLC	183.225 V	600.2141W	97.921%	Less	0.481 s	0.521 s	High	More
Utilization of 2nd Shading effect on the sunlight intensity condition (1000 W/m^2^, 755 W/m^2^, plus 655 W/m^2^)								
AMPANC-PI	168.270 V	505.99W	94.119%	More	1.162 s	1.208 s	Low	Low
IRBFAC-PI	170.212 V	509.127W	94.876%	More	0.710 s	0.89 s	Low	Low
IGO-P&O	170.989 V	514.88W	95.212%	Medium	0.581 s	0.617 s	Medium	Medium
MGWM-AFLC	175.761 V	518.998W	96.881%	Less	0.531 s	0.54 s	High	More

### Analysis of MGWM-AFLC MPPT controller at 1000 W/m^2^, 755 W/m^2^, plus 655 W/m^2^

**Figure 14:**
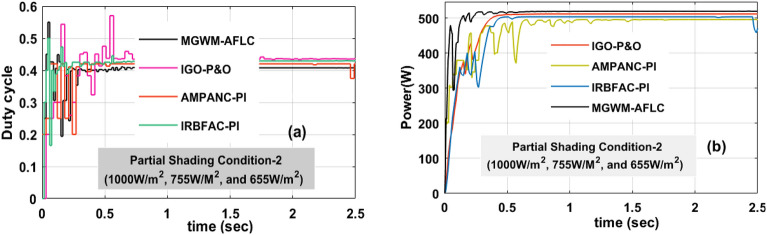
Introduced power boost DC-DC converter circuit, (**a**) duty signal, plus (**b**) DC-DC power at 2nd shading of sunlight system.

In this condition, the solar modules captured sunlight insolation’s are still reduced due to the cloudy atmospheric conditions. Here, the overall irradiation value is much less when associated with the previous two sunlight intensity conditions. The generated converter power and its related functioning duty values are mentioned in Fig. 14b, plus Fig. 14a. The evaluated converter circuit efficiencies, settling time, output power, plus voltages by integrating the AMPANC-PI, IRBFAC-PI,IGO-P&O, plusMGWM-AFLC MPPT methodologies are 94.119%, 1.208 s, 505.99W, 168.270 V, 94.876%, 0.89 s, 509.127W, 170.212 V, 95.212%, 0.617 s, 514.88W, 170.989 V, 96.881%, 0.54 s, 518.998W, plus 175.761 V respectively.

## Experimental development of the converter circuit

**Figure 15:**
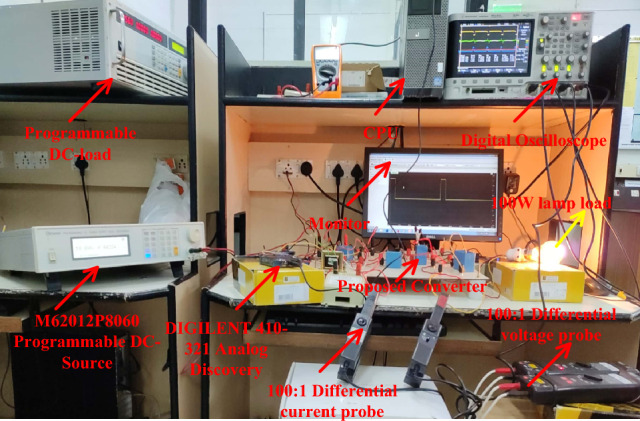
The utilized experimental power transfer converter circuit.

**Figure 16:**
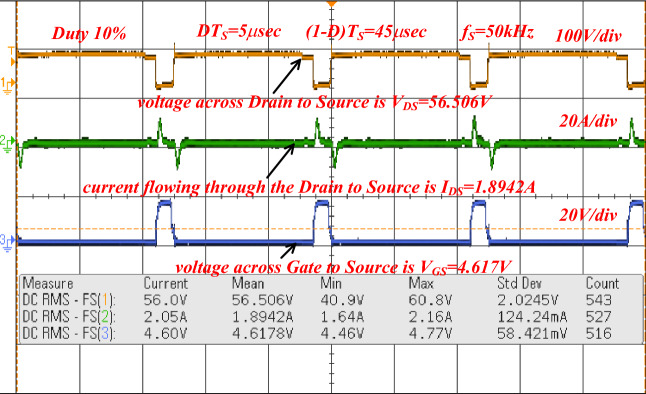
Voltages available across the power switches.

**Figure 17:**
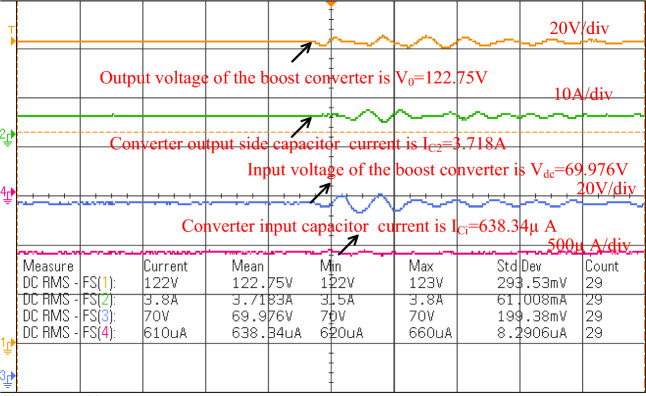
Overall power transfer circuit voltage waveforms.

The sunlight network power transfer ability is enhanced by selecting the power converter circuit. Here, the converter network is studied by utilizing the programmable power source. The overall testing set-up is mentioned in Fig. 15. The selected duty for enhancing the source supply voltage is 0.1 and its related frequency is 50 kHz. To turn on the switch (IRF-840N), the applied gate voltage is 4.6 V and its drain voltage is 56.5 V as given in Fig. 16. Here, the power source converter voltage value is 69.98 V, and it increased to 122.78 V from the switching pulses production of the TLP-350 driver board. The analog discovery helps operate the power converter circuit at less heating power losses. In the proposed power converter circuit, there are a few elements interfaced between the supply and load for filtering the distortions in the converter load voltage as given in Fig. 17. In this network, the major designed elements are TKD-film capacitors (C_u_, C_y_, C_r_, C_p_, plus C_0_) which are equal to 45µF. The utilized inductor elements for designing the circuit are L_m_, plus L_1_ which are equal to 250µH.

## Conclusion

The Modified Grey Wolf Methodology with Adaptive FLC for sunlight network is developed by using the MATLAB/Simulink tool. Here, in the first objective, the 3-diode sunlight module is designed to limit the drawbacks of reverse leakage currents, plus junction recombination power losses. This diode circuit functioning efficiency, plus fill factor are more when associated with the other PV models. The sunlight system drawback is less power production. The MGWM-AFLC is introduced to maintain the operating point of the sunlight system at peak power position thereby improving the power extraction ability of the solar system. This controller features fewer number iterations needed for identifying suitable membership values, optimal duty value selection for converter, less dependence on the sunlight system modeling, plus fast MPP tracking speed. Also, the solar network supply voltage is low. To enhance the voltage rating of the solar, the WPRHVBC power conversion network is proposed as the third objective for improving the load voltage of the system. The merits of this converter are low voltage stress of power switches, wide voltage gain, easy understanding, plus more suitable for shaded conditions of the sunlight systems.

## Data Availability

The datasets used and/or analyzed during the current study are available from the corresponding author upon reasonable request.

## Abbreviations

PV: Photovoltaic
MPP: Maximum power point
MPPT: Maximum power point tracking controller
GWM: Grey wolf methodology
AFLC: Adaptive fuzzy logic controller
MLPFFN: Multilayer perceptron feed-forward neural network
RBFN: Radial basis functional network
SMO: Sliding mode optimization
CPC: Conventional power converter
QSSPC: Quasi single switch power converter
USDSC: Universal source dual switch converter
HVCRC: High voltage conversion ratio converter
P&O: Perturb & observe
MHC: Modified hill climb
I_Dp_, I_D0_, I_Dy_, I_Du_: Converter diode currents
V_Dp_, V_D0_, V_Dy_, V_Du_: Converter diode voltages
I_Cp_, I_C0_, I_Cy_, I_Cu_: Capacitive currents
V_Cp_, V_C0_, V_Cy_, V_Cu_: Capacitive voltages
PSO: Particle swarm optimization
CSA: Cuckoo search algorithm
WVSC: Wide voltage source converter
IZSPC: Interleaved Z-source power converter
ITPPC: Interleaved three phase power converter
SSLSC: Single switch large source converter
